# Protectins PCTR1 and PD1 Reduce Viral Load and Lung Inflammation During Respiratory Syncytial Virus Infection in Mice

**DOI:** 10.3389/fimmu.2021.704427

**Published:** 2021-08-19

**Authors:** Katherine H. Walker, Nandini Krishnamoorthy, Thayse R. Brüggemann, Ashley E. Shay, Charles N. Serhan, Bruce D. Levy

**Affiliations:** ^1^Pulmonary and Critical Care Medicine, Department of Medicine, Brigham and Women’s Hospital and Harvard Medical School, Boston, MA, United States; ^2^Center for Experimental Therapeutics and Reperfusion Injury, Department of Anesthesiology, Perioperative and Pain Medicine, Brigham and Women’s Hospital and Harvard Medical School, Boston, MA, United States

**Keywords:** resolution, interferon, pro-resolving, docosahexaenoic acid, viral

## Abstract

Viral pneumonias are a major cause of morbidity and mortality, owing in part to dysregulated excessive lung inflammation, and therapies to modulate host responses to viral lung injury are urgently needed. Protectin conjugates in tissue regeneration 1 (PCTR1) and protectin D1 (PD1) are specialized pro-resolving mediators (SPMs) whose roles in viral pneumonia are of interest. In a mouse model of Respiratory Syncytial Virus (RSV) pneumonia, intranasal PCTR1 and PD1 each decreased RSV genomic viral load in lung tissue when given after RSV infection. Concurrent with enhanced viral clearance, PCTR1 administration post-infection, decreased eosinophils, neutrophils, and NK cells, including NKG2D^+^ activated NK cells, in the lung. Intranasal PD1 administration post-infection decreased lung eosinophils and *Il-13* expression. PCTR1 increased lung expression of cathelicidin anti-microbial peptide and decreased interferon-gamma production by lung CD4^+^ T cells. PCTR1 and PD1 each increased interferon-lambda expression in human bronchial epithelial cells *in vitro* and attenuated RSV-induced suppression of interferon-lambda in mouse lung *in vivo*. Liquid chromatography coupled with tandem mass spectrometry of RSV-infected and untreated mouse lungs demonstrated endogenous PCTR1 and PD1 that decreased early in the time course while cysteinyl-leukotrienes (cys-LTs) increased during early infection. As RSV infection resolved, PCTR1 and PD1 increased and cys-LTs decreased to pre-infection levels. Together, these results indicate that PCTR1 and PD1 are each regulated during RSV pneumonia, with overlapping and distinct mechanisms for PCTR1 and PD1 during the resolution of viral infection and its associated inflammation.

## Introduction

**Figure 1:**
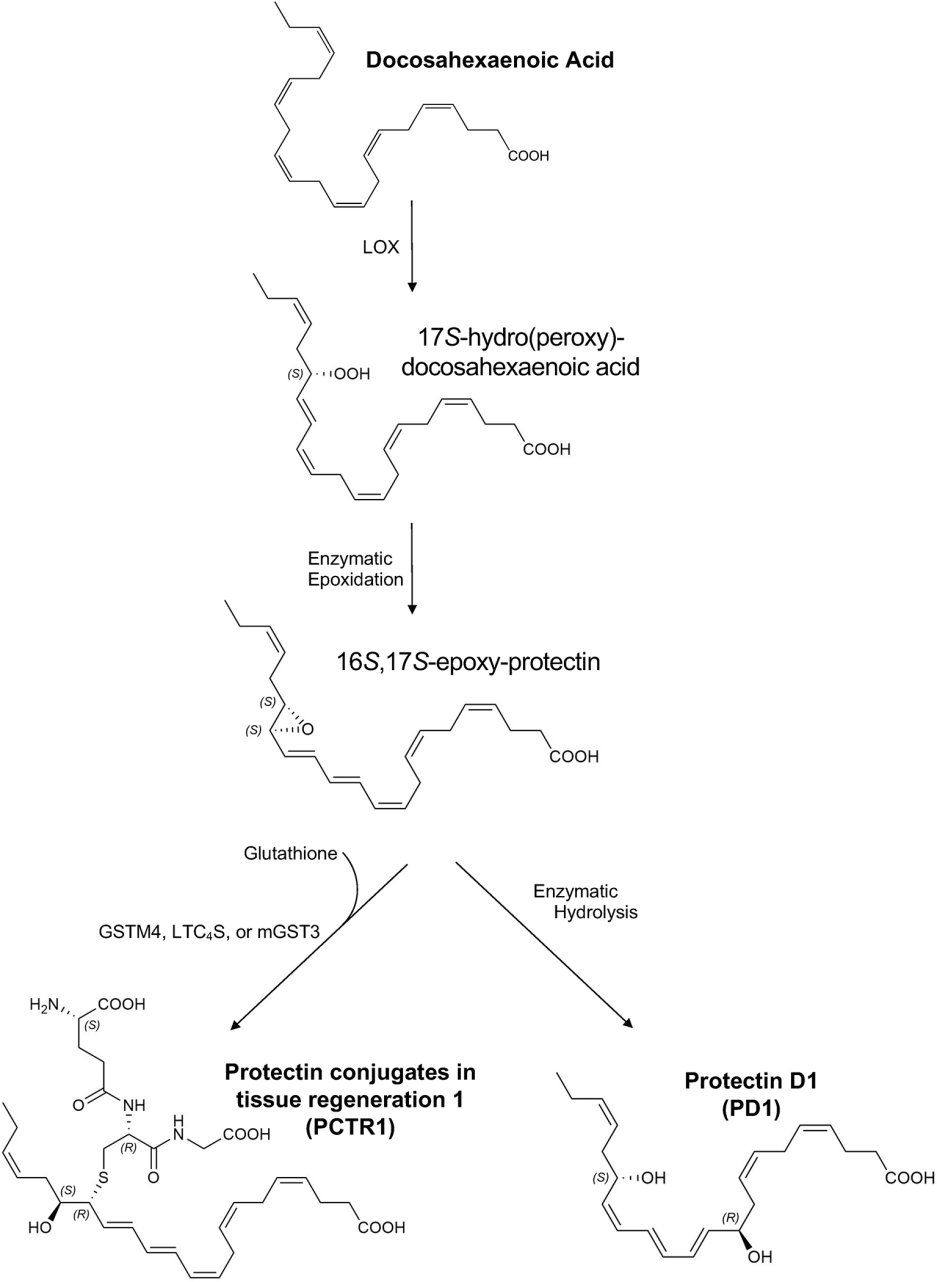
Proposed biosynthesis of protectin conjugates in tissue regeneration 1 (PCTR1) and protectin D1 (PD1). Docosahexaenoic acid (DHA) is converted to 17*S*-hydro(peroxy)-docosahexaenoic acid (17*S*-H(p)DHA) *via* 15-lipoxygenase (LOX) in human cells and 12/15-LOX in mice (5, 6). Enzymatic epoxidation of 17*S*-H(p)DHA produces the 16*S*,17*S*-epoxy-protectin intermediate (5, 6). Enzymatic hydrolysis of the 16*S*,17*S*-epoxy-protectin intermediate generates protectin D1 (PD1) (5, 6) and glutathione S-transferase-mediated (LTC_4_S, leukotriene C_4_ synthase; GSTM4, glutathione S-transferase Mu 4; mGST3, microsomal glutathione S-transferase 3) S_N_2 reaction with glutathione produces protectin conjugates in tissue regeneration 1 (PCTR1) (4, 7, 8). The products and intermediates are depicted in their assigned complete stereochemical configurations (4–6).

The resolution of inflammation is an actively regulated process in which specialized pro-resolving mediators (SPMs) play a prominent role. SPMs are endogenous lipid mediators that include lipoxins, resolvins, maresins, and protectins, which are enzymatically produced from essential polyunsaturated fatty acids (1). SPMs can drive the resolution of acute inflammatory processes to restore tissue homeostasis after sterile or infectious injury and can enhance pathogen clearance in animal infection models (2, 3). Of particular interest is the docosahexaenoic acid (DHA)-derived protectin family of SPMs, including protectin conjugates in tissue regeneration 1 (PCTR1: 16*R*-glutathionyl, 17*S*-hydroxy-4*Z*,7*Z*,10*Z*,12*E*,14*E*,19*Z*-docosahexaenoic acid) (4) and protectin D1 (PD1: 10*R*,17*S*-dihydroxy-4*Z*,7*Z*,11*E*,13*E*,15*Z*,19*Z*-docosahexaenoic acid) (5) (see **Figure 1**).

DHA is converted to 17*S*-hydro(peroxy)-docosahexaenoic acid (17*S*-H(p)DHA) *via* 15-lipoxygenase (LOX) in human cells and 12/15-LOX in mice (5, 6). Enzymatic epoxidation of 17*S*-H(p)DHA produces the 16*S*,17*S*-epoxy-protectin intermediate (5, 6). Enzymatic hydrolysis of the 16*S*,17*S*-epoxy-protectin intermediate generates protectin D1 (PD1) (5, 6) and glutathione S-transferase-mediated (LTC_4_S, leukotriene C_4_ synthase; GSTM4, glutathione S-transferase Mu 4; mGST3, microsomal glutathione S-transferase 3) S_N_2 reaction with glutathione produces protectin conjugates in tissue regeneration 1 (PCTR1) (4, 7, 8). Because of structural similarities between PCTRs and other cysteinyl-SPMs (cys-SPMs) such as maresin conjugates in tissue regeneration, PCTR1 is proposed to be subsequently converted to PCTR2 by γ-glutamyl transferase, followed by conversion to PCTR3 by dipeptidases (9). Of note, this biosynthetic pathway can be influenced by neuronal stimuli such as acetylcholine (10). Protectins act through G-coupled receptors (11) and intracellular mediators such as TNF receptor associated factor 3 (12).

Importantly, PCTR1 is present in human lung tissue (13) and decreases neutrophil migration while increasing macrophage recruitment and efferocytosis (4). PD1 is present in human exhaled breath condensates and decreases during acute airway inflammation such as asthma exacerbation (14); PD1 decreases T cell migration, promotes T cell apoptosis, and reduces inflammatory cytokine production (15).

Respiratory Syncytial Virus (RSV) is a leading cause of viral lower respiratory tract infection in children and elderly patients, causing over 34 million estimated infections and 3.5 million estimated hospitalizations annually across the globe (16, 17). The most severe cases are marked clinically by respiratory failure and pathologically by peribronchial and interstitial inflammation, with airways often occluded by cellular debris and mucus (18). The host response to RSV infection is complex, involving multiple cellular and molecular factors, overall promoting viral clearance but often causing significant pathogen-associated lung pathology (19). Although anti-inflammatory agents such as steroids suppress inflammation, they do not improve RSV outcomes and are limited by immunosuppressive risks including secondary bacterial infection and impaired viral clearance (20). While monoclonal antibodies can prevent severe RSV infection in high-risk infants (21), no therapies are available for active infection, making new approaches to RSV treatment urgently necessary (22).

The relationship between SPMs and viral-mediated acute lung inflammation is of interest. PCTR1 promotes resolution of bacterial inflammation (4); the role of PCTR1 in viral infection remains to be determined. During influenza infection, strain virulence is associated with impaired SPM signaling in mice, and PD1 limits viral replication *via* inhibition of viral RNA nuclear export *in vitro*, improving survival when administered *in vivo* in a mouse model (23). During RSV infection, enzymatic activity of 5-lipoxygenase, necessary for biosynthesis of the lipoxin and resolvin families of SPMs, promotes resolution of lung injury *via* alternative activation of macrophages (24); however, the roles of PCTR1 and PD1 from the protectin pathway remain to be determined in RSV infection.

Here, we investigated PCTR1 and PD1’s actions in RSV infections and identified temporal regulation of protectins in the lung after RSV infection. When given post-infection, these protectins attenuated lung inflammation, reduced viral burden, and regulated interferon and anti-microbial peptide expression.

## Materials and Methods

### Viral Propagation and Quantification

Respiratory Syncytial Virus, strain Line 19, was originally isolated from a sick infant and generously provided by Dr. Nicholas Lukacs (University of Michigan, MI). Virus was propagated in HEp-2 cells (ATCC, Manassas, VA) and quantified by plaque assay, similar to prior publications (25) using NY3.2 STAT1^-/-^ murine fibroblast cells (26) generously provided by Dr. Jennifer Bomberger (University of Pittsburgh, PA). Cells were cultured at 37°C in 5% CO_2_ in Dulbecco’s Modified Eagle’s Medium (DMEM) with L-glutamine (Lonza, Mapleton, IL) supplemented with 10% Fetal Bovine Serum (Denville Scientific, Metuchen, NJ), 100 U/mL penicillin with 100 μg/mL streptomycin (Lonza), and 10 μg/mL gentamycin (Sigma-Aldrich, St. Louis, MO).

### Animals and Infection

Experimental protocols were approved by the Institutional Animal Care and Use Committee at Harvard Medical School/Brigham and Women’s Hospital. 6-8 week old C57BL/6 male mice (Jackson Laboratory, Bar Harbor, ME) were housed in specific pathogen-free conditions at 25°C with regular light and dark cycles at the Center for Comparative Medicine at Brigham and Women’s Hospital. Mice were fed *ad libitum* with a standard commercial diet including approximately 13.1% total fat and 0.5% omega-3 fatty acids (ScottPharma, Marlborough, MA). Under inhaled isoflurane anesthesia, mice were infected with 10^5^ Plaque Forming Units (PFU) of viral stock in 30-50 µL of Hep2 cell culture media. In some experiments, isoflurane-anesthetized mice were administered 15 µL of SPM (100 ng) or vehicle (Phosphate Buffered Saline, PBS) intranasally (i.n.) on days 3, 4, 5 post infection (p.i.) and harvested on day 6 or day 8 p.i.

### Specialized Pro-Resolving Mediators

PCTR1 (16*R*-glutathionyl, 17*S*-hydroxy-4*Z*,7*Z*,10*Z*,12*E*,14*E*,19*Z*-docosahexaenoic acid), ^13^ ${\text{C}}_{2}^{15}$N-PCTR1, and ^13^ ${\text{C}}_{3}^{15}$N-MCTR3 were each synthesized by total organic synthesis and provided by Dr. Nicos A. Petasis (University of Southern California) *via* subcontract for P01GM095467 to CNS. PCTR1, PD1 (10*R*,17*S*-dihydroxy-4*Z*,7*Z*,11*E*,13*E*,15*Z*,19*Z*-docosahexaenoic acid), 17-HDHA, d_4_-LTB_4_, d_8_-5*S*-HETE, d_5_-LTC_4_, d_5_-LTD_4_, LTC_4_, LTD_4_, and LTE_4_ were purchased from Cayman Chemical (Ann Arbor, MI). For cellular administration, PCTR1, PD1, or vehicle (ethanol) was diluted in media to a final ethanol concentration <0.1%. For mouse administration, PCTR1, PD1, or vehicle (ethanol) was brought to dryness with a gentle stream of nitrogen gas prior to resuspension in PBS. Before experiments, each SPM was authenticated and validated using an unbiased library search (Sciex OS Version 1.7.0.36606) and was in accordance with its reported physical properties established earlier (4, 5).

### Quantitative Polymerase Chain Reaction

Perfused left lungs or cell monolayers were homogenized in TRIzol (Invitrogen, Carlsbad, CA) and total RNA was extracted using a chloroform extraction method as previously described (27). After DNAse treatment (Invitrogen), cDNA was generated using the Taqman Reverse Transcription Kit (Thermo Fisher Scientific, Waltham, MA). Samples were quantified on AriaMx real-time qPCR system (Agilent Technologies, Santa Clara, CA) using EvaGreen supermix (Bio-Rad, Hercules, CA) and primers as described (Integrated DNA Technologies, Coralville, IA) (**Table S1**). Quantification was performed using the 2^-ΔΔCt^ method, using *18s* housekeeping gene to calculate fold change relative to naïve control or RSV-infected vehicle control as specified. Mouse and human mRNA expression of the PD1 receptor GPR37 (11) in sorted lung cells was obtained from the public LungMAP Database (https://lungmap.net).

### Histopathology

Right lungs were fixed by inflation with Zinc Fixative (BD Biosciences) *via* tracheostomy at a transpulmonary pressure of 20 cm H_2_O. Lung sections were stained by the Rodent Histopathology Core at Harvard Medical School. Images were obtained using a CX33 microscope with EP50 camera (Olympus Life Science, Tokyo, Japan).

### Lung Cell Preparation

Lungs were perfused with 5mL PBS *via* right ventricular puncture, extracted and maintained on ice in PBS with 0.7 mg/mL Collagenase A (Roche, Cambridge MA), 30 μg/mL DNase I (Sigma-Aldrich) and 2% FBS. Lung tissue was dissociated into a single cell suspension using a gentleMACS dissociator (Miltenyi Biotec, Somerville, MA) according to the manufacturer’s instructions. Lung cells were filtered through a 70 µm strainer (Thermo Fisher Scientific) and counted in Trypan Blue (Sigma-Aldrich) *via* hemocytometer (eFluor 506, Thermo Fisher Scientific). Cells were stained with viability dye (eFluor 506, Thermo Fisher Scientific), fixed and permeabilized using the FoxP3 kit (eBioscience San Diego, CA) according to the manufacturer’s instructions prior to staining for flow cytometry. In some cases, cells were stimulated in a commercial cocktail of phorbol 12-myristate 13-acetate, ionomycin, brefeldin A and monensin (Tonbo Biosciences, San Diego CA) for 4 hours prior to viability dye, fixing, permeabilization, and staining.

### Flow Cytometry

The following antibodies were used to identify cellular subsets and intracellular cytokines in whole lung homogenates: from BioLegend (San Diego, CA) CD45-PerCP, CD11c-FITC, Ly6G-AF700, MHC class II-BV421, CD4-FITC, CD8a-APC-Cy7, CD3-Q655, NKG2D-PE, NK1.1-PE-Cy7, IFNγ-PE; from eBioscience, CD11b-APC; from BD Biosciences (San Jose, CA) SiglecF-PE. Samples were obtained using an LSR Fortessa flow cytometer (BD Biosciences) and analyzed using FlowJo software version 10 (Ashland, OR). Cells of interest were identified after exclusion of debris, dead cells and doublets (**Figure S2**).

### Bronchoalveolar Lavage and Enzyme-Linked Immunosorbent Assay (ELISA)

Lungs were lavaged with two separated volumes of 1 mL each of 0.6 mM EDTA in PBS, *via* an intratracheal catheter. ELISA for murine interferon-alpha (PBL Assay Science, Piscataway, NJ), interferon-beta and interferon-lambda 2/3 (R&D Systems, Minneapolis, MN) in BAL samples was performed according to manufacturer’s instructions. Interferon levels were normalized to total protein content in BAL fluid as measured by bicinchoninic acid assay (Thermo Fisher Scientific).

### Human Airway Epithelial Cells

Calu-3 (ATCC) and A549 (ATCC) human airway epithelial cell lines were cultured in Eagle’s Minimum Essential Media (ATCC) or Dulbecco's Modified Eagle's Medium (Lonza), respectively, with 10% FBS, penicillin, and streptomycin as above. Cell monolayers were grown to >80% confluency, washed with PBS and infected in media with 0% FBS at a multiplicity of infection of 0.05 or 0.1 for 2 hours. Viral inoculum was replaced by 10% FBS media with PCTR1 or PD1 (10 nM) or vehicle (ethanol) and cells were incubated at 37°C in 5% CO_2_ for 24 hours.

### Lipid Mediator Metabololipidomics

Un-perfused lung tissue was snap-frozen in liquid nitrogen, prior to addition of ice-cold liquid chromatography/mass spectrometry-grade methanol (Thermo Fisher Scientific) containing 500 pg of each of the following deuterium-labeled internal standards: d_8_-5*S*-HETE (Cayman Chemical), d_4_-LTB_4_ (Cayman Chemical), d_5_-LTC_4_ (Cayman Chemical), d_5_-LTD_4_ (Cayman Chemical), ^13^ ${\text{C}}_{3}^{15}$N-MCTR3, and ^13^ ${\text{C}}_{2}^{15}$N-PCTR1 for calculating extraction and recovery of endogenous material. Lungs were gently dispersed using a glass tissue grinder (Kimble Chase Life Science and Research Products, Vineland, NJ) and protein precipitation occurred at -20°C for 30 minutes. Lung suspensions were centrifuged at 1000 g for 10 minutes at 4°C, supernatants were collected, and products were solid phase extracted per optimized methods using an automated extractor (Extrahera, Biotage, Charlotte, NC) as in reference (8). Samples were brought to an apparent pH 3.5 with acidified water (9 mL), and rapidly loaded onto 3 mL-SPE Isolute C18 100 mg cartridges (Biotage) and neutralized with double-distilled water (4 mL). The columns were washed once with hexane (Supelco, Bellefonte, PA) (4 mL). Next, the methyl formate fraction (Sigma-Aldrich) (4 mL) eluted SPMs, prostaglandins, leukotrienes, and thromboxane. The methanol fraction (Thermo Fisher Scientific) (4 mL) eluted cys-SPMs and cys-LTs. Both the methyl formate and methanol fractions were separately brought to dryness with a gentle stream of nitrogen gas using an automated evaporation system (TurboVap LV, Biotage), and immediately suspended in a methanol-water mixture (50:50, *v/v*) for injection on liquid chromatography tandem mass spectrometer (LC-MS/MS). Samples were injected and data acquired using a LC-MS/MS 6500^+^ QTRAP in low mass mode (Sciex, Framingham, MA) equipped with an ExionLC (Shimadzu, Tokyo, Japan).

A Kinetex Polar C18 column (100 mm x 4.6 mm x 2.6 µm; Phenomenex, Torrance, CA, USA) was kept in a column heat jacket maintained at 50°C. (**Table S2**) specifies polarity, retention time (min), Q1 (*m/z*), Q3 (*m/z*), dwell time (msec), declustering potential (DP, V), entrance potential (EP, V), collision energy (CE, V), collision cell exit potential (CXP, V), calibration correlation coefficient (r^2^), and lower limit of detection (LLOD, pg) for each mediator. The mobile phase gradient, multiple reaction monitoring (MRM), and enhanced product ion (EPI) mode settings are described in (**Table S3**). For each mediator, linear calibration curves were obtained using synthetic material with r^2^ values of ≥0.98. Identification of each mediator included unbiased MS/MS matching (>70% fit score) to authentic and synthetic material in a MS/MS library (library matching parameters: precursor mass tolerance ± 0.8 Da, fragment mass tolerance ± 0.4 Da, collision energy ± 5 eV, use polarity, intensity threshold = 0.05, minimal purity = 5.0%, and intensity factor = 100) and a matching retention time to those of the authentic and synthetic material. Data was acquired with Analyst 1.7.1 software (Sciex). The MS/MS spectral library was created in LibraryView version 1.4.0 (Sciex). LC-MS/MS MRM trace data and EPI spectral data are shown as screen captures from Sciex OS version 1.7.0.36606 (Sciex).

### Statistics

Statistical analysis was performed using GraphPad Prism software, version 9 (San Diego, CA) or RStudio (Boston, MA). One-way ANOVA with Holm-Sidak’s correction for multiple comparisons was used for parametric data, and Kruskall-Wallis test with Dunn’s correction for multiple comparisons was used for non-parametric data. Findings were considered significant when *p≤*0.05 and not significant when *p*>0.10. Statistical outliers were excluded by Rout’s outlier analysis (Q = 1%). For qPCR experiments, statistical analysis was performed on ΔCT values for time-course data, and on ΔCT values normalized to each experiment (ΔCT of sample/average ΔCT of the RSV-infected vehicle control group) for replicate experiments of RSV-infected mice.

## Results

### RSV Pneumonia Resolves Spontaneously in C57/BL6 Mice

**Figure 2:**
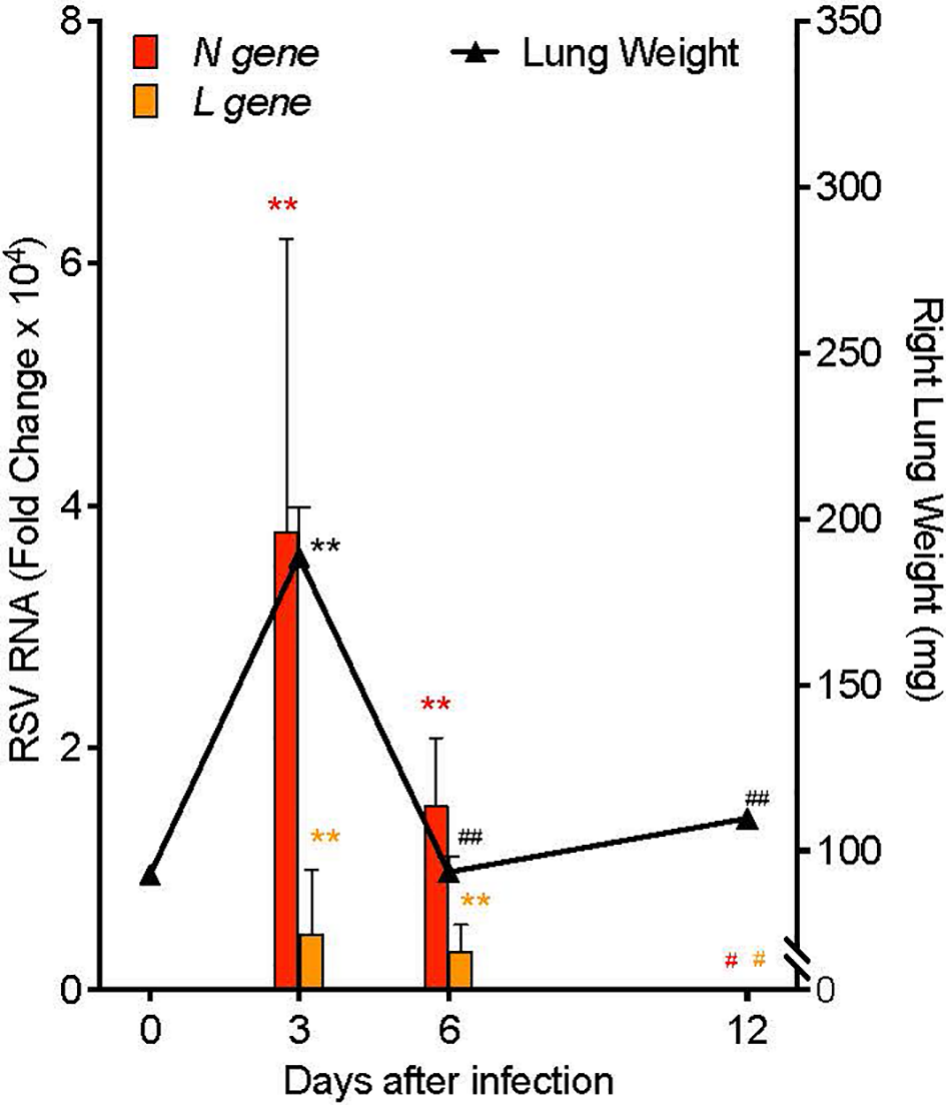
Mouse RSV pneumonia resolves spontaneously. Mice were infected with 10^5^ PFU of the RSV Line19 strain. Left axis: RSV *N gene* and *L gene* viral RNA expression in left lung tissue. Right axis: weight of right lung. Values are mean of n=3-4 per time point ± SEM and are representative of 2 separate experiments. **p < 0.01 vs Day 0, ^#^ *p ≤ * 0.05 vs Day 3, ^##^ *p* < 0.01 vs Day 3 by One-way ANOVA with Holm-Sidak’s multiple comparison correction.

In order to investigate mechanisms of resolution of RSV infection and associated inflammation, we generated a self-limited RSV pneumonia model: C57/BL6 mice were infected intranasally with 10^5^ Plaque Forming Units (PFU) of a clinically-isolated strain of RSV, Line 19 (28). Lung weight and RNA copies of RSV genes were measured serially after infection (**Figure 2**). Weight of the right lung was significantly increased 3 days post infection (p.i.) (189.0 ± 15.1 mg) compared to pre-infection lung weight (92.6 ± 2.0 mg, *p*<0.001) and to lung weights at 6 days p.i. (93.5 ± 4.9 mg, *p*<0.001) and 12 days p.i. (110.0 ± 1.78 mg, *p*<0.001). Simultaneously, viral load, as measured by quantitative PCR (qPCR) of the RSV nucleoprotein (*N*) gene RNA in whole left lung, was increased day 3 p.i. (mean fold change of 3.79 ± 2.41 x10^4^, *p*=0.010) and day 6 p.i. (mean fold change of 1.52 ± 0.56 x10^4^, *p*=0.010), with no remaining difference by day 12 p.i. Similarly, RNA copies of the RSV Large polymerase (*L*) gene, which correlate with active viral replication (29), were increased at day 3 p.i. (mean fold change of 4.63 ± 2.66 x10^3^, *p*=0.001) and day 6 p.i. (mean fold change of 3.27 ± 1.05 x10^3^, *p*=0.001), with no remaining difference by day 12 p.i.

### PCTR1 and PD1 Decrease Viral Burden During RSV Infection

**Figure 3:**
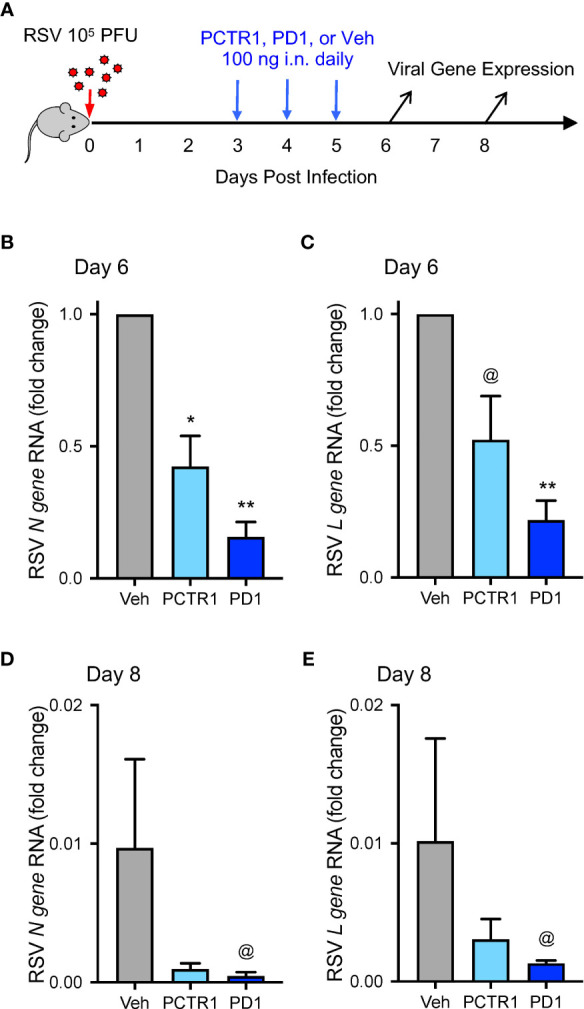
PCTR1 and PD1 decrease viral transcripts during mouse RSV infection. **(A)** Schema of experimental mouse infection with intranasal 10^5^ PFU of RSV, administration of PCTR1 or PD1 (100 ng intranasal) or vehicle daily on days 3-5 post infection, and measurement of viral gene expression on day 6 and day 8. **(B–E)** Relative RNA expression of **(B)** RSV *N gene* or **(C)** RSV *L gene* in the mouse lung tissue at day 6 after RSV infection or of **(D)** RSV *N gene* or **(E)** RSV *L gene* at day 8. Values are mean of n=4-9 per group ± SEM and include >4 separate experiments **(B, C)** or 1 experiment **(D, E)**. *p < 0.05, **p < 0.01, ^@^ *p ≤ * 0.10 vs vehicle by Kruskall-Wallis test with Dunn’s multiple comparison correction.

Because PCTR1 and PD1 are present in lung tissue (13, 14) and PD1 reduced viral burden of influenza (23), we evaluated whether PCTR1 or PD1 could facilitate host resolution of RSV infection in this model. C57/BL6 mice were infected intranasally (i.n.) with 10^5^ PFU of RSV as above, prior to i.n. treatment with PCTR1 (100 ng), PD1 (100 ng), or vehicle on days 3, 4, and 5 p.i., and lungs were harvested at day 6 or day 8 p.i. (**Figure 3A**). RSV *N gene* RNA was significantly decreased in PCTR1 and PD1 cohorts (mean fold changes of 0.40 ± 0.11 and 0.21 ± 0.10, respectively; *p*=0.032 and *p*<0.001, respectively) relative to vehicle control 6 days after RSV infection (**Figure 3B**). In addition, RSV *L gene* RNA transcripts were also decreased in PCTR1- and PD1-exposed lungs (mean fold changes of 0.68 ± 0.29 and 0.21 ± 0.07, respectively; *p*=0.057 and *p*=0.004, respectively) compared to vehicle control at day 6 p.i. (**Figure 3C**). These trends continued at day 8 p.i., with lower viral RNA transcripts of RSV *N gene* and RSV *L gene* in lung tissue of PCTR1 and PD1 cohorts compared to vehicle control. At day 8 relative to day 6 p.i., the vehicle mean fold change was 0.010 ± 0.006 for RSV *N* gene and 0.010 ± 0.007 for RSV *N gene* (**Figures 3D, E**). PCTR1 and PD1 treatment each further decreased RSV *N gene* viral transcripts on day 8 relative to vehicle control (mean fold change 0.001 ± <0.001 and <0.001 ± <0.001, respectively; *p*=0.191 and *p*=0.062, respectively) (**Figure 3D**). Similarly, PCTR1 and PD1 treatment each also decreased RSV *L gene* transcripts on day 8 relative to vehicle control (mean fold changes 0.003 ± 0.001 and 0.001 ± <0.001, respectively; *p*=0.653 and *p*=0.100) (**Figure 3E**).

Of note, mRNA copies of the recently identified RSV entry receptor, Insulin-like Growth Factor 1 Receptor (*Igf1r*), were not significantly changed after PCTR1 or PD1 exposure in mice at day 6 p.i. (ΔCT values of 13.9 ± 0.5 and 13.6 ± 0.7 respectively, compared to 13.6 ± 0.4 for vehicle control; *p*=ns for both). Calu-3 human airway epithelial cells express the PD1 receptor gene *GPR37* (11) (**Figure 5F**). Exposure to PCTR1 or PD1 did not significantly change RSV *N gene* or RSV *L gene* copies (**Figure S1**) or *IGF1R* expression (ΔCT values of 11.9 ± 0.1 and 11.8 ± 0.4 respectively, compared to 11.1 ± 0.7 for vehicle control; *p*=ns for both) in directly infected Calu-3 airway epithelial cells *in vitro* at 24h p.i. The human airway epithelial cell line A549 also expressed *GPR37* RNA and was infected similarly without significant changes in viral gene expression after exposure to PCTR1 or PD1 (ΔCT values of 7.7 ± 0.1 and 7.9 respectively, compared to 8.1 ± 0.3 for vehicle control; *p*=ns for both).

### PCTR1 and PD1 Decrease Lung Inflammation During RSV Infection

**Figure 4:**
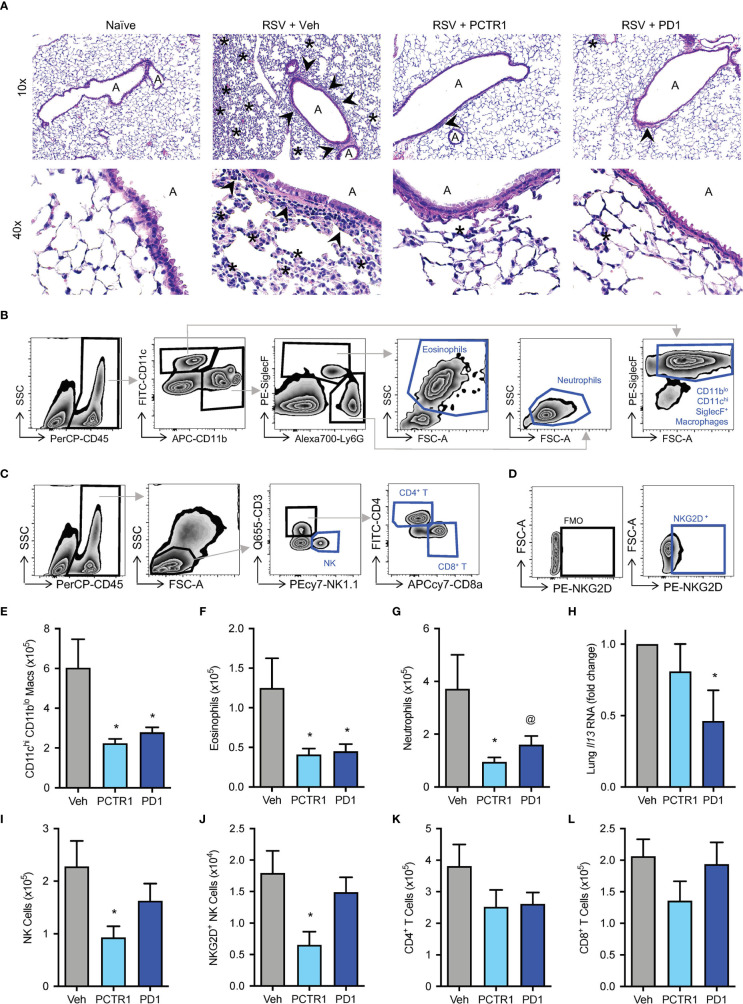
PCTR1 and PD1 decrease inflammatory responses to mouse RSV infection. Mice were infected with 10^5^ PFU of RSV, followed by administration of PCTR1 or PD1 (100 ng intranasal) or vehicle daily on days 3-5 post infection and harvest of lungs at day 6. **(A)** Representative hematoxylin- and eosin-stained histology of mouse lung tissue at 10x and 40x magnification. “A” identifies airway lumen; arrowheads denote areas of peri-bronchiolitis; Asterix denotes area of interstitial alveolitis. **(B, C)** Gating strategy for identification of **(B)** macrophage and granulocyte or **(C)** lymphocyte subsets, after doublet and dead cell exclusion. **(D)** The NKG2D^+^ NK cell population was determined by comparison to Fluorescence Minus One (FMO) staining. **(E–G)** Populations of **(E)** CD11c^hi^ CD11b^lo^ macrophages, **(F)** Eosinophils and **(G)** Neutrophils per right lung by flow cytometry. **(H)** Relative *Il-13* RNA expression in lung tissue. **(I–L)** Populations of **(I)** NK cells, **(J)** NKG2D^+^ NK cells, **(K)** CD4^+^ T cells, **(L)** CD8^+^ T cells per right lung by flow cytometry. Values are mean of n ≥ 4 per group ± SEM and include >4 separate experiments **(B, C, E–I, K, L)** or 2 separate experiments **(D, J)**. *p < 0.05 vs vehicle, ^@^ *p* < 0.10 vs vehicle by one-way ANOVA with Holm-Sidak’s multiple comparisons correction.

Given the decreases in viral load with *in vivo* PCTR1 or PD1 treatment, we evaluated effects of these SPMs on leukocyte responses to RSV. Mice were treated as in **Figure 3A**. At day 6 p.i., representative histology revealed alveolitis and peribronchial inflammation in RSV-infected mice exposed to vehicle control, with attenuation of these findings in infected mice exposed to PCTR1 or PD1 (**Figure 4A**). Given the predominantly interstitial nature of lung inflammation observed in this C57/BL6 mouse model, we evaluated single-cell lung homogenates by flow cytometry analysis for quantification of cellular subtypes (**Figures 4B–D**, **Figure S2**). Compared to vehicle, PCTR1 and PD1 significantly decreased lung CD11c^hi^CD11b^lo^ macrophages (2.24 ± 0.22 and 2.79 ± 0.26, respectively, vs 6.03 ± 1.44 cells x10^5^ [vehicle], *p*=0.012 and 0.019) (**Figure 4E**) and lung eosinophils (0.41 ± 0.08 and 0.45 ± 0.09, respectively, vs 1.25 ± 0.37 cells x10^5^ [vehicle], *p*=0.041 for both) (**Figure 4F**). PCTR1 significantly decreased lung neutrophils (0.95 ± 0.17 vs 3.72 ± 1.29 cells x10^5^ [vehicle], *p*=0.044), with a similar trend for PD1 (1.60 ± 0.33 cells x10^5^, *p*=0.082 vs vehicle) (**Figure 4G**).

Because skewing of cytokine and adaptive immune responses from type 1 to type 2 is associated with more severe RSV disease (29, 30), we evaluated the type 2 gene *Il-13* mRNA transcripts. *Il-13* expression was significantly decreased in infected mice exposed to PD1 relative to vehicle control (fold change 0.47 ± 0.22 vs vehicle, *p*=0.037) (**Figure 4H**). No significant changes in *Il-13* expression were present in mice exposed to PCTR1. Of note, neither PCTR1 nor PD1 increased *Il-13* expression or eosinophilia (**Figures 4F, H**).

Additionally, PCTR1 decreased the number of Natural Killer (NK) cells in the lung (0.93 ± 0.21 vs 2.28 ± 0.49 cells x10^5^ [vehicle], *p*=0.023) (**Figure 4I**), including NK cells expressing the NKG2D activating receptor (0.65 ± 0.21 vs 1.79 ± 0.35 cells x10^4^ [vehicle], *p*=0.033) (**Figure 4J**), with a similar trend for CD4^+^ and CD8^+^ T cells (**Figures 4K, L** **)**.

### PCTR1 and PD1 Regulate Host Antiviral Responses During RSV Infection

**Figure 5:**
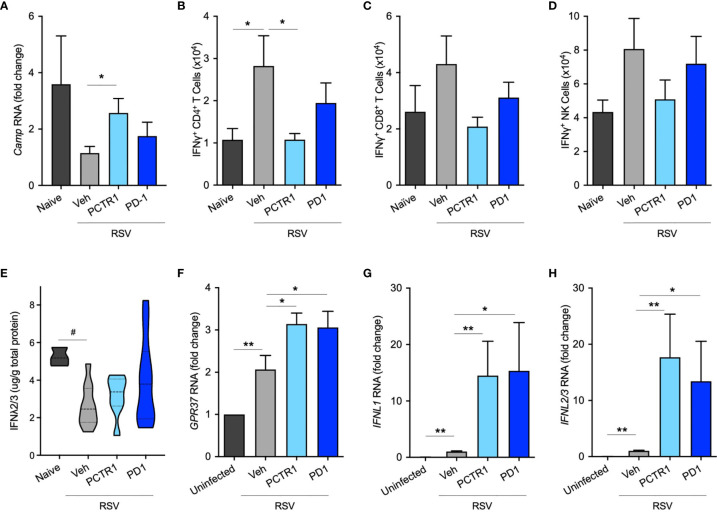
PCTR1 and PD1 regulate host antiviral responses after RSV infection in mice. **(A)** Cathelicidin Anti-Microbial Peptide (*Camp)* RNA expression in mouse lung tissue at day 6 post infection with RSV 10^5^ PFU. **(B–D)** Intracellular IFNγ expression in **(B)** CD4^+^ T, **(C)** CD8^+^ T, and **(D)** NK cells. **(E)** IFNλ2/3 protein concentrations in bronchoalveolar lavage fluid. **(F–H)** Relative RNA expression of **(F)** *GPR37*, **(G)** *IFNL1*, and **(H)** *IFNL2/3* in human airway epithelial cells. Values are mean of n = 4-11 per RSV group, n = 3-4 per naïve or uninfected group, ± SEM and include ≥3 separate experiments **(A–E)** or 2 separate experiments **(F–H)**. *p ≤  0.05, **p < 0.01 by one-way ANOVA with Holm-Sidak’s multiple comparisons correction; ^#^ *p ≤* 0.05 by Kruskall-Wallis test with Dunn’s multiple comparisons correction.

Since PCTR1 and PD1 decreased viral load and inflammatory parameters *in vivo*, we next investigated whether PCTR1 or PD1 regulated anti-microbial peptide expression or interferon (IFN) signaling as potential mechanisms for enhanced viral clearance distinct from cellular inflammation. Expression of murine Cathelicidin Anti-Microbial Peptide (*Camp*, the human ortholog of which is also called *LL-37*) was significantly increased in the PCTR1 cohort compared to vehicle (fold change 2.48 ± 0.51, *p*=0.036) (**Figure 5A**). As measured by intracellular flow cytometric staining of stimulated CD4^+^ T lymphocytes, PCTR1 significantly decreased interferon-gamma (IFNγ) expression compared to vehicle, as measured by IFNγ^+^ CD4^+^ T cells (1.08 ± 0.15 vs 2.82 ± 0.72 x10^4^, *p*=0.029), returning expression to pre-infection baseline (**Figure 5B**), with a similar trend for IFNγ^+^ CD8^+^ T cells and IFNγ^+^ NK cells (**Figures 5C, D**). Concurrent with attenuation of IFNγ production, PCTR1 and PD1 increased expression of interferon-lambda (IFNλ) during RSV infection. RSV infection significantly decreased IFNλ protein levels in bronchoalveolar lavage (BAL) fluid relative to naïve controls (2.64 ± 0.35 [RSV + vehicle] vs 5.23 ± 0.28 [naïve] µg/g total protein, *p*=0.029). Of note, PCTR1 and PD1 prevented the RSV-induced decreases in IFNλ, maintaining the protective levels of IFNλ concentrations as those of uninfected mice (3.96 ± 0.68 and 3.15 ± 0.42 µg/g, respectively, *p*=ns for both) (**Figure 5E**). Type I interferons, interferon-alpha (IFNα) and -beta (IFNβ), were undetectable in BAL fluid at day 6 p.i.

In human airway epithelial cells directly infected with RSV, RNA expression of the PD1 receptor gene, *GPR37*, increased during RSV infection (2.07 ± 0.33 fold change compared to uninfected controls, *p*=0.007) and increased further with PCTR1 or PD1 exposure (fold changes 3.14 ± 0.26 and 3.06 ± 0.38, respectively, vs uninfected control, p<0.001 for both) (**Figure 5F**). Of note, RNA sequencing data from the LungMAP database (31) indicate that *GPR37* is expressed by epithelial cells in lung tissue throughout development in mice and humans (**Figure S3**). Exposure of RSV-infected human airway epithelial cells to either PCTR1 or PD1 significantly increased RNA expression of two isoforms of IFNλ, *IFNL1* (mean fold increases of 14.51 ± 6.07 and 15.35 ± 8.54, respectively, *p*=0.010 and *p*=0.016) and *IFNL2/3* (mean fold increases of 17.70 ± 7.64 and 13.43 ± 7.10, *p*=0.003 and *p*=0.016) compared to the RSV-infected vehicle cohort (**Figures 5G, H**). mRNA transcripts of *IFNα* and *IFNβ* were not detectable in these samples 24 hours after infection.

### PCTR1 and PD1 Axes Are Temporally Regulated During RSV Infection

Since exogenous PCTR1 and PD1 administered on day 3 p.i. led to reduced genomic viral load and decreased interstitial lung inflammation when measured on day 6 p.i., we next evaluated whether endogenous production of protectins was altered during this self-limited model of RSV infection. Mice were infected with 10^5^ PFU of RSV and lung tissue was subjected to multiple reaction monitoring (MRM) by liquid chromatography coupled with tandem mass spectrometry (LC-MS/MS) for evaluation of targeted lipid mediators at specified timepoints over the course of RSV infection.

**Figure 6:**
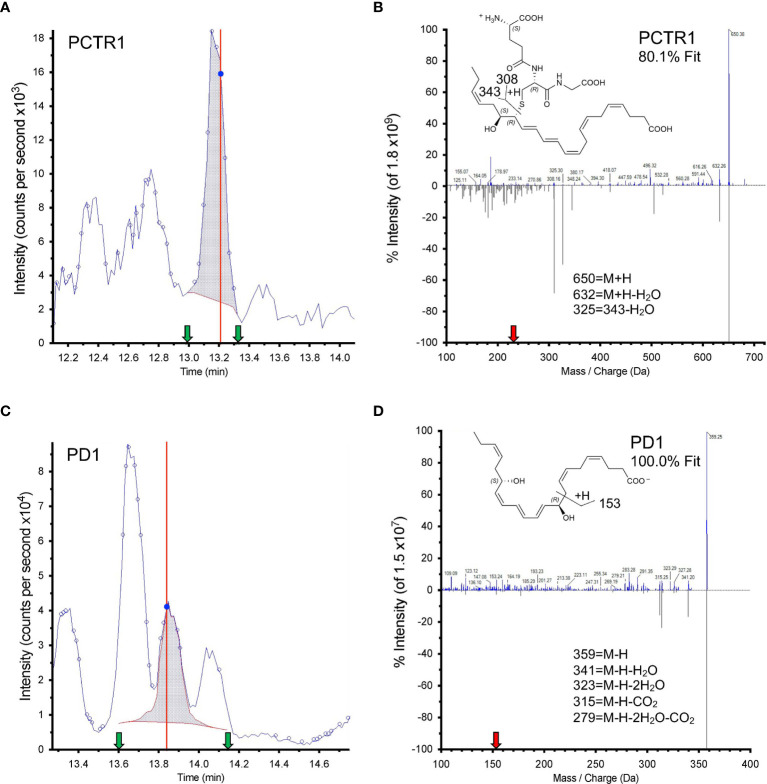
Identification of PCTR1 and PD1 from RSV-infected mouse lung. **(A, C)** Screen captures from Sciex software of **(A)** PCTR1 and **(C)** PD1 targeted multiple reaction monitoring (MRM). Dark blue data-point indicates where the spectra for **(B, D)** were collected; vertical red line indicates the retention time for that data point. Shaded blue area denotes the area under the curve used for quantitation. Green arrows denote retention time at start and finish of quantitation. **(B, D)** Structure and tandem mass spectrometry fragmentation for **(B)** PCTR1 (*m/z* 650>231) with 80.1% unbiased fit to library and matching retention time and **(D)** PD1 (*m/z* 359>153) with 100% unbiased fit to library and matching retention time, analyzed with Sciex software. Upper MS/MS denotes sample spectrum; mirrored lower MS/MS denotes library spectrum obtained for synthetic PCTR1 and PD1. The red arrow on the x axis indicates Q3 used for quantitation. Insets show structures and proposed ion fragments.

Both PCTR1 and PD1 were detected in naïve and RSV-infected lung tissue in mice (**Figures 6A, C**), identified by an 80.1% unbiased library fit score and a 100.0% unbiased library fit score, respectively, as well as matching retention times to authentic and synthetic material (**Figures 6B, D**). PCTR1 and PD1 were significantly decreased at day 3 p.i. compared to naïve control (1.3 ± 0.8 pg/50 mg of lung tissue vs 12.4 ± 1.6 for PCTR1, *p*=0.001; 14.3 ± 1.4 vs 49.1 ± 3.7 for PD1, *p*=0.007) (**Table 1**) and increased significantly by day 12 p.i. (17.5 ± 0.9 pg/50 mg of lung tissue and 52.8 ± 3.7, respectively, compared to day 3 values; *p*<0.001 and *p*=0.004) (**Table 1**). Identification of the PD1 isomer protectin Dx (PDx, 10*S*,17*S*-dihydroxy-4*Z*,7*Z*,11*E*,13*Z*,15*E*,19*Z*-docosahexaenoic acid) and the separation between PD1 and PDx was also confirmed by unbiased library matching and retention time matching to authentic and synthetic material (data not shown). PCTR2 and PCTR3 were not detected in lungs during mouse RSV infection. Of interest, the DHA lipoxygenase product 17-hydroxy-docosahexaenoic acid (17-HDHA), a marker of the protectin biosynthetic pathway (**Figure 1**), was also significantly decreased at day 3 p.i. compared to naïve control (355.5 ± 46.2 pg/50 mg of lung tissue vs 1152.1 ± 61.9, respectively, *p*=0.004) and significantly increased by day 12 p.i. (1711.9 ± 128.7 pg/50 mg of lung tissue, *p*<0.001 compared to day 3 p.i.) (**Table 1**).

**Table 1:** Members of the protectin pathway of SPMs are temporally regulated by RSV infection in mice.

pg/50 mg tissue	Naïve	Day 3 p.i.	Day 6 p.i.	Day 12 p.i.
PCTR1	12.4 ± 1.6	1.3 ± 0.8*	8.3 ± 2.0^#^	17.5 ± 0.9
PD1	49.1 ± 3.7	14.3 ± 1.4*	31.0 ± 8.6	52.8 ± 3.7^#^
17-HDHA	1152.1 ± 61.9	355.5 ± 46.2*	965.0 ± 168.3^#^	1711.9 ± 128.7^#^

Mice were infected with 10^5^ PFU of RSV and lung was obtained at the specified time points post infection (p.i.). Values are mean of n = 3-4 mice per time point ± SEM. *p < 0.01 vs naïve, ^#^p < 0.01 vs day 3 post-infection by one-way ANOVA with Holm-Sidak’s multiple comparisons correction.

**Figure 7:**
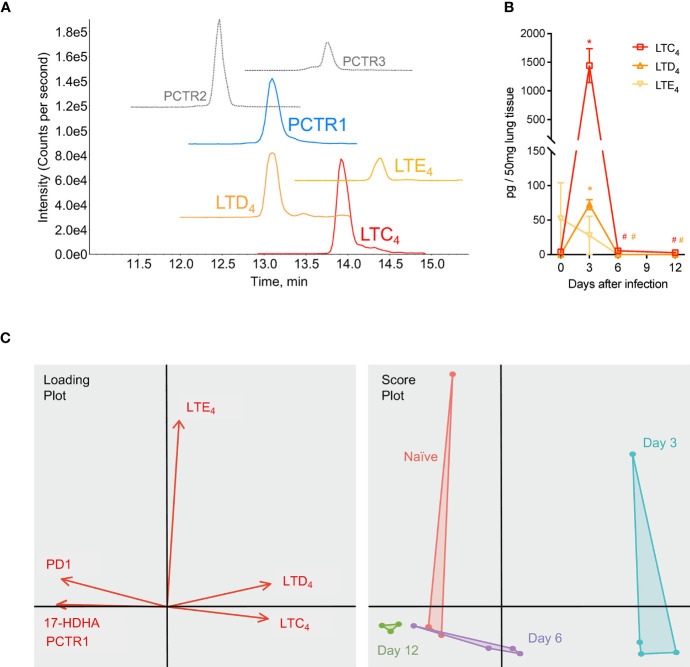
Protectins and cysteinyl-leukotrienes (cys-LTs) are temporally regulated during mouse RSV infection. Mice were infected with 10^5^ PFU of RSV and right lung was harvested at the time points stated. **(A)** Targeted multiple reaction monitoring (MRM) for PCTR1-3, LTC_4_, LTD_4_, and LTE_4_ (1.0-point Gaussian smoothed) synthetic material, using Sciex ExionLC and 6500^+^ Triple Quadrupole QTRAP operated in positive mode (see **Table S3** for details). **(B)** Time course of cys-LTs from RSV infections; data from LC-MS/MS quantitation expressed as pg/50 mg of mouse lung tissue. **(C)** Principal component analysis plots for PCTR1, PD1, 17-HDHA, LTC_4_, LTD_4_, and LTE_4_ in lung tissues of naïve mice (red) or mouse lung tissues at day 3 (blue), day 6 (purple), and day 12 (green) post-infection with RSV. Values are mean of n = 3-4 mice per group ± SEM. *p < 0.01 vs naïve, ^#^ *p* < 0.01 vs day 3 post-infection by one-way ANOVA with Holm-Sidak’s multiple comparisons correction.

In addition to members of the protectin pathway, cys-LTs were also identified and detected in mouse lung tissue by LC-MS/MS and targeted MRM for each product (**Figure 7A**). Leukotriene C_4_ (LTC_4_) and D_4_ (LTD_4_) significantly increased in lung tissue at day 3 p.i. compared to that of naïve mice (1441 ± 296 pg/50 mg of lung tissue vs 3.74 ± 1.87 for LTC_4_, *p*<0.001; 72.50 ± 7.25 vs 0.03 ± <0.01 for LTD_4_, *p*<0.001) (**Figure 7B**). Both mediators significantly decreased by day 6 p.i. (5.48 ± 0.45 pg/50 mg lung tissue and 0.03 ± <0.01, respectively; *p*=0.001 and *p*<0.001 for each compared to day 3 p.i. values) (**Figure 7B**). Leukotriene E_4_ was not significantly changed over the course of infection. As visualized in a Principal Components Analysis, the array of protectin and leukotriene mediators (PCTR1, PD1, 17-HDHA, LTC_4_, LTD_4_, and LTE_4_) deviated from naïve baseline at day 3 p.i. and returned towards baseline over the subsequent time points during the resolution of infection (**Figure 7C**).

**Figure 8:**
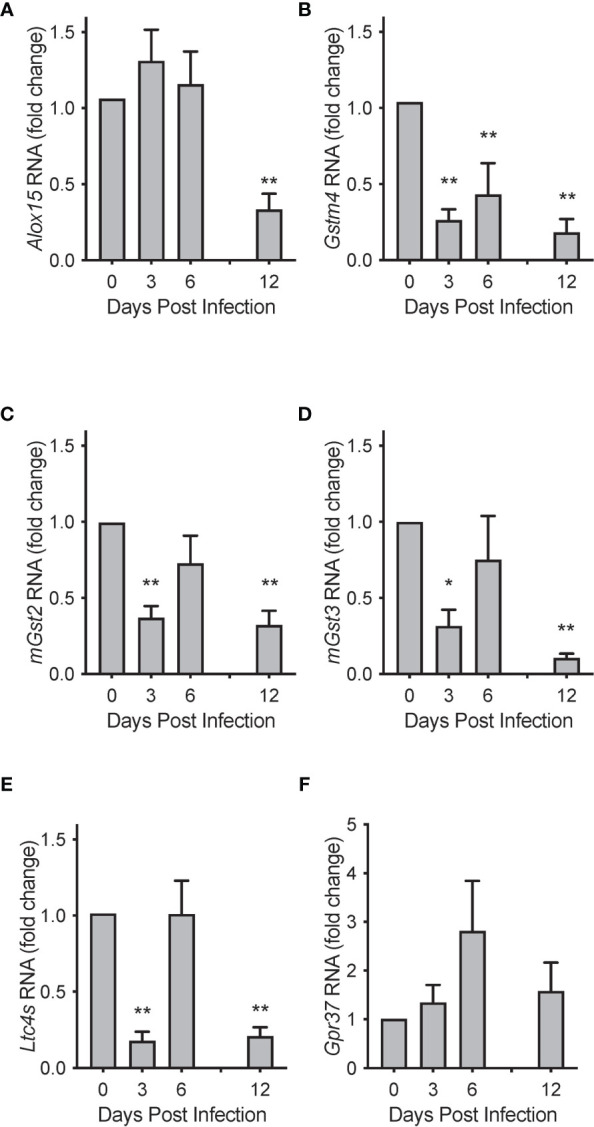
Biosynthetic enzymes for protectins are temporally regulated during mouse RSV infection. Relative RNA expression of **(A)** *Alox15*, **(B)** *Gstm4*, **(C)** *mGst2*, **(D)** *mGst3*, and **(E)** *Ltc_4_s* in mouse lung tissue over the course of RSV infection, compared to naïve control. **(F)** Protectin D1 receptor *Gpr37* relative RNA expression in mouse lung tissue over the course of RSV infection. Values are mean of n = 3-4 per group ± SEM and represent 2 separate experiments. *p < 0.05, **p < 0.01 vs naïve by one-way ANOVA with Holm-Sidak’s multiple comparisons correction.

To investigate potential mechanisms for these temporal changes in SPM concentrations during RSV infection, we evaluated mRNA transcript copies of enzymes known to catalyze the production of PCTR1 and PD1 from DHA (**Figure 1**). Lung mRNA expression of 15-Lipoxygenase (gene *Alox15*) was not significantly changed at days 3 or 6 p.i. (mean fold changes of 1.30 ± 0.41 and 1.20 ± 0.43 compared to naïve control, *p*=0.255 and *p*=0.223, respectively) but was significantly reduced at 12 days p.i. (mean fold change 0.33 ± 0.10 compared to naïve control, *p*<0.001) in the setting of viral clearance (**Figure 8A**). We next explored RNA expression of identified glutathione S-transferases that convert epoxy intermediates to PCTR1 and LTC_4_ (8). *Gstm4* transcripts were significantly decreased at day 3, day 6, and day 12 p.i. (mean fold changes of 0.26 ± 0.07, 0.43 ± 0.20, and 0.18 ± 0.09 respectively compared to naïve control, *p*=0.003 for all) (**Figure 8B**). RNA expression of *mGst2* was suppressed to a lesser extent at day 3 and day 12 p.i. (mean fold changes of 0.37 ± 0.08 and 0.32 ± 0.09 compared to naïve control, *p*=0.009 and *p*=0.007 respectively) (**Figure 8C**). *mGst3* transcripts were significantly decreased in mouse lung tissue at day 3 and day 12 p.i. (mean fold changes of 0.032 ± 0.11 and 0.11 ± 0.03 compared to naïve control, *p*=0.015 and *p*<0.001 respectively) (**Figure 8D**). *Ltc_4_s* was significantly decreased to a similar extent as *Gstm4* at day 3 and day 12 p.i. (mean fold changes of 0.18 ± 0.06 and 0.21 ± 0.06, respectively, compared to naïve control; *p*<0.001 for both) (**Figure 8E**).

Expression of the PD1 receptor gene *Gpr37* (11) did not change significantly over the 12 days of infection (**Figure 8F**). RNA sequencing data from the LungMAP database (31) suggests that, within murine lung, *Gpr37* is most abundantly expressed on epithelial and mesenchymal cells by 4 weeks of age (**Figure S3A**) and in humans, persists in epithelial cells throughout life (**Figure S3B**).

## Discussion

Here, exogenous PCTR1 and PD1 decreased viral burden of RSV and concurrently decreased inflammation. Specifically, PCTR1 and PD1 decreased lung eosinophils, neutrophils and macrophages, with PCTR1 also decreasing both total and NKG2D^+^ activated NK cells. This attenuated inflammation was associated with decreased *Il-13* expression with PD1, decreased IFNγ and increased anti-microbial peptide expression with PCTR1 *in vivo*, and increased IFNλ expression with either PCTR1 or PD1 *in vitro*. Of interest, endogenous levels of PCTR1 and PD1 were noted to be decreased in the early phase of RSV infection, with associated decreases in biosynthetic enzyme expression, during a concurrent increase in cys-LTs. These changes in lung lipid mediator profiles were most divergent from baseline at day 3 p.i., returning to baseline levels by day 12.

Since PCTR1 (13) and PD1 (14) are present in lung tissue and PD1 has been noted to suppress propagation of influenza virus (23), we examined the viral response to exogenous PCTR1 or PD1 and found that each molecule decreased genomic viral load during RSV infection *in vivo*, without evidence of a delay in viral clearance. Of interest, we did not find evidence of direct inhibition of viral replication in an *in vitro* model of airway epithelial cell infection with RSV, as has been observed with PD1 and PDx during influenza infection (23, 32). This difference suggested that innate and/or adaptive immune responses were relevant to PCTR1- or PD1-mediated control of RSV.

Immune responses to RSV are necessary for viral clearance but can also cause significant immunopathology (19). The pathology of fatal cases of human RSV infection suggests that host responses to limit viral replication also contribute to injury of ‘innocent bystander’ lung tissue, with inflammation present despite lack of detectable viral antigen in some cases (18). At a cellular level, NK cells, CD8^+^ T cells, CD4^+^ T cells and eosinophils have been implicated in promoting viral clearance and exacerbating pathogen-initiated lung injury in murine models of RSV infection (19). Here, PCTR1 and PD1 were associated with decreased lung inflammation and decreased viral burden, suggesting pro-resolving rather than immunosuppressive mechanisms. Both PCTR1 and PD1 decreased lung granulocytes and alveolar macrophages, and PCTR1 reduced both total and activated NK cells in the lung, suggesting that control of viral burden was not due to an exuberant inflammatory response. A similar trend was seen with CD4^+^ T and CD8^+^ T cells, possibly reflecting later timing of the adaptive immune response in this model, as has been appreciated with similar models (19). Additionally, building on the identification of PD1 decreases in IFNγ secretion from human T cells *ex vivo* (15), the reduction of IFNγ expression in CD4^+^ and CD8^+^ T cells observed here suggests that PCTR1, and to some extent PD1, regulated type 1 inflammation in this model. This attenuation – rather than obliteration – of type 1 inflammation is important because knock-out and early neutralization experiments suggest that resolution of RSV infection depends on IFNγ signaling and leukocyte responses (33, 34). The regulation of inflammation by PCTR1 and PD1 – without evidence of immunosuppression – highlights a unique property, differentiating these protectins from current anti-inflammatory therapies for RSV that are clinically limited by infectious risks (35, 36).

Both clinical studies of hospitalized infants and mechanistic studies of infected mice have shown that skewing of the immune response from type 1 cytokines (including IFNγ) to type 2 cytokines (such as IL-4 and IL-13) is associated with severe RSV-induced immunopathology (19, 29, 30, 33). Of note, the decreased type 1 inflammation seen in this model was not related to skewing of the immune response to type 2 inflammation because lung eosinophils were suppressed by PCTR1 and PD1; moreover, lung *Il-13* expression was stable or decreased. Notably, PD1 has been shown to be produced by T helper type 2 skewed leukocytes in human blood (15), supporting a possible negative feedback mechanism for type 2 inflammation in this model. Decreases in neutrophils and macrophages observed in the lungs of mice treated with PCTR1 or PD1 suggest against skewing of the immune response towards IL-17 mediated immunity, as well. Of importance in models of non-infectious lung inflammation, SPMs promote resolution by multiple mechanisms (36). PCTR1 promotes the resolution of tissue injury by decreasing neutrophil infiltration and pro-inflammatory cytokines while enhancing macrophage phagocytosis, efferocytosis and planaria tissue regeneration (4). In murine models, PD1 reduces granulocyte migration into areas of inflammation, decreases pro-inflammatory cytokine production and enhances macrophage efferocytosis (2, 14, 15). Together, these provide possible mechanisms for the lower granulocyte counts seen in these PCTR1- and PD1-treated mice.

The concurrent decreases in both viral burden and lung inflammation, without evidence of direct inhibition of RSV replication in epithelial cells, suggested that mucosal host defense mechanisms may play a role in PCTR1- and PD1-mediated *in vivo* actions to promote the resolution of RSV infection and inflammation. Indeed, PCTR1 increased expression of *Camp*. This action adds to prior findings that members of the lipoxin family of SPMs induce lung *Camp* expression during infection (3). Cathelicidins can attenuate RSV infections by directly damaging the virus envelope, hence decreasing viral binding and cellular entry, and promoting an antiviral state in surrounding airway epithelial cells (37). PCTR1 induction of *Camp* in this model would thus be expected to enhance viral host defense. Of note, cathelicidins can induce pro-inflammatory leukotriene production (38); however, PCTR1 decreased the inflammatory response to RSV at day 6 p.i. in this model. Cathelicidin binds to the lipoxin receptor ALX/FPR2 to induce LTB_4_ production from human neutrophils, yet SPMs can effectively interrupt this mechanism for leukotriene induction to control inflammatory responses (3, 38).

Type I interferons (primarily IFNα, IFNβ) and type III interferons (isoforms of IFNλ) also promote an antiviral state through intracellular signaling cascades leading to induction of interferon-stimulated genes. While the type I interferon receptor is present on many cell types including leukocytes, IFNλ’s effects are limited to those cells expressing its heterodimeric receptor: primarily epithelial cells. This differential receptor expression may contribute to the particular importance of IFNλ to airway mucosal host responses, with IFNλ less likely to promote inflammation than IFNα/β (39, 40) but still able to reduce RSV infection of epithelial cells (40). The ability of RSV proteins to suppress these antiviral interferons is an important component of RSV pathogenicity, with more severe infant RSV infections associated with relatively lower expression of interferon-related genes (41). The RSV NS1, NS2 and G proteins can suppress IFNα and IFNβ production and signaling *via* multiple mechanisms (19); RSV NS1 and NS2 proteins suppress IFNλ expression (40) and the RSV F protein has been implicated in EGFR-mediated suppression of IFNλ production (42).

Here, PCTR1 and PD1 increased IFNλ expression during RSV infection of human airway epithelial cells and each mediator blunted RSV-mediated suppression of murine IFNλ *in vivo*. Of interest, IFNλ has been noted to enhance macrophage phagocytosis and efferocytosis as well as drive macrophage stimulation of NK cell and CD8^+^ T cell cytotoxicity (43) – all critical components of host anti-viral responses. Indeed, PCTR1 and PD1 increase macrophage phagocytosis in other models (2, 4). These data and prior literature suggest possible mechanisms for PCTR1 and PD1 control of viral burden in the airway without exacerbating inflammation. Additionally, combined with the identification of Protectin D1 receptor *GPR37* expression in mouse lung tissue and human airway epithelial cells (31) and PD1 effects noted on airway epithelial cells *in vitro* (23), this regulation suggests that PCTR1 and PD1 act directly on airway epithelial cells during viral infection. This induction of IFNλ by protectins appears to be the first evidence for endogenous inducers of host IFNλ expression. While IFNα and IFNβ were undetectable at day 6 p.i. of this model, it is possible that these interferons could also be regulated by PD1 or PCTR1 at earlier timepoints, thereby facilitating the viral clearance seen at day 6 p.i.

Given these actions of PCTR1 and PD1, we investigated whether endogenous levels of these SPMs were affected by RSV infection. While PD1 is transiently decreased during influenza infection in mouse lung (23), the impact of other viral infections on PD1 synthesis in the lung – and of any viral infection on PCTR1 synthesis – has been unknown. Here, mRNA expression of the enzymes for PCTR1 and PD1 synthesis was suppressed by RSV infection, and levels of protectins in the lung were transiently decreased, ultimately increasing concurrent with RSV clearance.

RSV Long strain induces mRNA expression of *Alox15*, the first enzyme in the conversion of DHA to PCTR1 or PD1, at days 1-4 post infection in mouse lungs (24). Here, using the Line 19 strain of RSV, we observed steady *Alox15* expression 3-6 days after infection, followed by a significant decrease in expression at day 12 p.i. In contrast to *Alox15* expression, we observed significantly decreased mRNA transcripts of *Ltc_4_s*, *mGst3*, and *Gstm4* – biosynthetic enzymes downstream of *Alox15* for PCTR1 synthesis – when viral titers were highest at day 3 p.i. These transcriptional changes and the decreased lung tissue concentrations of protectins suggest that the biosynthetic pathway for protectins was suppressed by RSV in this model.

Of interest, leukotriene synthesis was increased at day 3 p.i. compared to naïve control. This increase in cys-LTs despite decreases in mRNA expression of rate-limiting enzymes (*Ltc_4_s*, *mGst2*, *mGst3*, or *Gstm4*) may have been secondary to persistent enzyme function after initial changes in mRNA transcripts or changes in mediator degradation. Additionally, lipid mediator levels are subject to spatial regulation of biosynthetic enzymes relative to substrate availability and to differences in enzyme-substrate affinity (8). Expression of *Gpr37*, a cellular receptor for PD1 (11), was not significantly changed over the course of RSV infection. Together, these data suggest that the signaling pathways thus far identified for PCTR1 and PD1 are temporally regulated during RSV infection. Notably, the pattern of temporal regulation of PCTR1 and PD1 observed here during RSV infection appears similar to that of PD1 during influenza infection (23), suggesting a possible shared mechanism for viral host responses.

RSV is an important human pathogen for which there are currently no effective treatments. While this mouse model faithfully replicates aspects of human RSV infection including interstitial mononuclear alveolitis, peribronchial inflammation and eosinophilia, it does not incorporate all features of human infection (18). Further determination of relationships between protectins and interferons will involve translation to human subjects in ongoing studies.

In conclusion, PCTR1 and PD1 engaged host protective mechanisms and decreased lung inflammation in this RSV pneumonia model. These each stimulated decreased viral burden, notably without apparent host immunosuppression, suggesting pro-resolving more than anti-inflammatory actions. As such, PCTR1 and PD1 may serve as investigational tools to better understand both the pathogenesis of viral lung infection and the possibility of harnessing host pro-resolving mechanisms for new therapeutic benefits.

## Data Availability Statement

The original contributions presented in the study are included in the article/**Supplementary Material**. Further inquiries can be directed to the corresponding authors.

## Ethics Statement

The animal study was reviewed and approved by Institutional Animal Care and Use Committee at Harvard Medical School.

## Author Contributions

KW conceived of the study, designed experiments, performed experiments, analyzed data and wrote and edited the manuscript. NK designed experiments, performed experiments, analyzed data, supervised the study and edited the manuscript. TB performed experiments, analyzed data and edited the manuscript. AS performed experiments, analyzed data and edited the manuscript. CS designed experiments, analyzed data and edited the manuscript. BL conceived of the study, designed experiments, analyzed data, supervised the study and edited the manuscript. All authors contributed to the article and approved the submitted version.

## Funding

This work was supported by National Institutes of Health F32AI134019-01 (to KW), Harvard Catalyst | The Harvard Clinical and Translational Science Center | National Center for Advancing Translational Sciences, National Institutes of Health Award UL 1TR002541 (to KW), T32HL007633-31 (to BL), and P01GM095467 (to CS and BL). The content is solely the responsibility of the authors and does not necessarily represent the official views of Harvard Catalyst, Harvard University and its affiliated academic healthcare centers, or the National Institutes of Health.

## Conflict of Interest

CS and BL are inventors on patents (protectins) assigned to Brigham and Women’s Hospital.

The remaining authors declare that the research was conducted in the absence of any commercial or financial relationships that could be construed as a potential conflict of interest.

## Publisher’s Note

All claims expressed in this article are solely those of the authors and do not necessarily represent those of their affiliated organizations, or those of the publisher, the editors and the reviewers. Any product that may be evaluated in this article, or claim that may be made by its manufacturer, is not guaranteed or endorsed by the publisher.

## Abbreviations

17-HDHA, 17-hydroxydocosahexaenoic acid; ANOVA, Analysis of Variance; BAL, Bronchoalveolar Lavage; CAMP, Cathelicidin Anti-Microbial Peptide (previously LL-37 in humans); cys-, cysteinyl; DHA, Docosahexaenoic Acid; EPI, Enhanced product ion; GSTM4, Glutathione S-transferase Mu 4; IFN, Interferon; IL, Interleukin; i.n., intranasal; LC-MS/MS, Liquid Chromatography coupled with tandem Mass Spectrometry; LOX, Lipoxygenase; LT, Leukotriene; LTC_4_S, Leukotriene C_4_ synthase; Ly6G, Lymphocyte antigen 6 complex locus G6D; mGST, microsomal glutathione S-transferase; NKG2D, Natural Killer Group 2D; NK1.1, Natural Killer cell marker, also known as Killer Cell Lectin-like Receptor subfamily B member (KLRB1C) or CD161; ns, not significant; PBS, Phosphate Buffered Saline; PCTR1, Protectin conjugates in tissue regeneration 1: 16*R*-glutathionyl, 17*S*-hydroxy-4*Z*,7*Z*,10*Z*,12*E*,14*E*,19*Z*-docosahexaenoic acid; PD1, protectin D1: 10*R*,17*S*-dihydroxy-4*Z*,7*Z*,11*E*,13*E*,15*Z*,19*Z*-docosahexaenoic acid; PDx, protectin Dx: 10*S*,17*S*-dihydroxy-4*Z*,7*Z*,11*E*,13*Z*,15*E*,19*Z*-docosahexaenoic acid; p.i., post infection; RSV, Respiratory Syncytial Virus; SiglecF, Sialic acid-binding immunoglobulin-like lectin F; SPM, Specialized pro-resolving mediator; Veh, Vehicle.
