# Supplementary material for: Protectins PCTR1 and PD1 Reduce Viral Load and Lung Inflammation During Respiratory Syncytial Virus Infection in Mice
Source: Front Immunol. 2021 Aug 19;12:704427. doi: 10.3389/fimmu.2021.704427 (PMC8417406; doi:10.3389/fimmu.2021.704427)
Supplement: Supplementary file 1 [file Presentation_1.pdf]

Supplemental information for

Protectins PCTR1 and PD1 Reduce Viral Load and Lung Inflammation during Respiratory Syncytial Virus Infection in Mice

Authors

Katherine H. Walker, Nandini Krishnamoorthy, Thayse R. Brüggemann, Ashley E. Shay, Charles N. Serhan, Bruce D. Levy

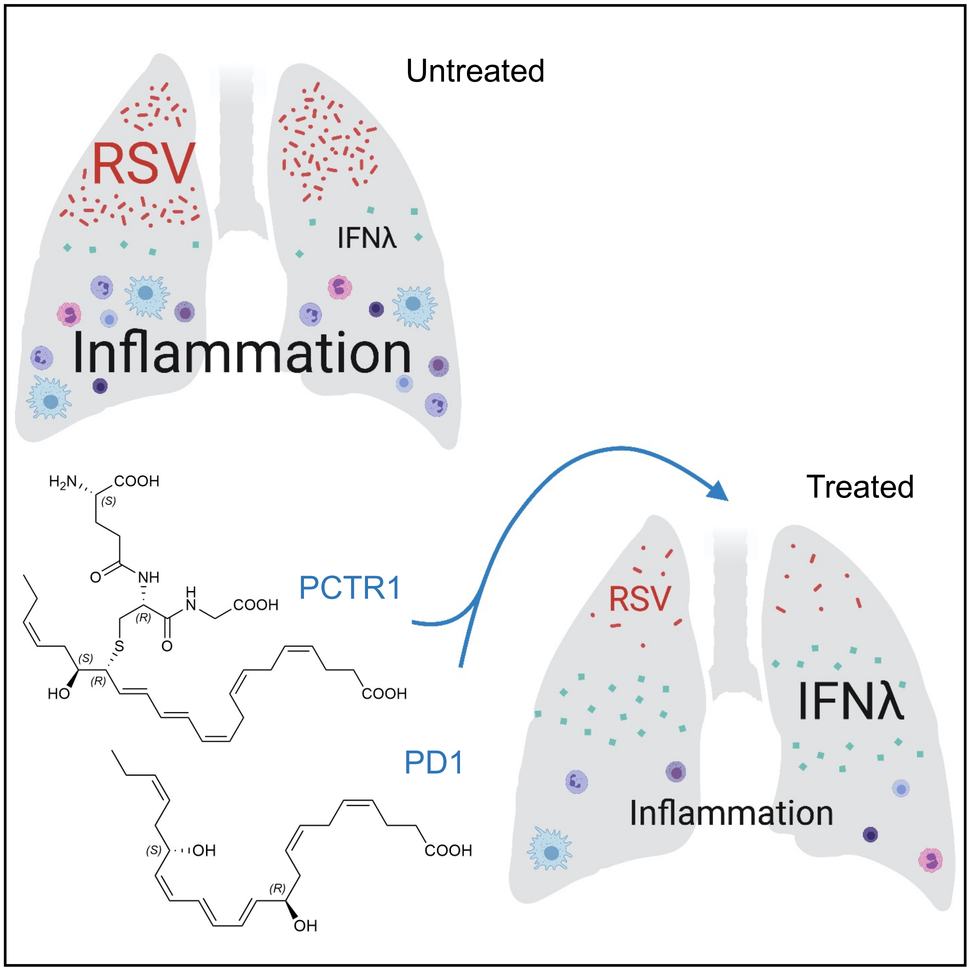

Gene	Species	Forward Primer, 3' → 5'	Reverse Primer, 3' → 5'
Alox15	M	CAGGGATCGGAGTACACGTT	GATTGTGCCATCCTTCCAGT
Lct4s	M	GCTCTTCTGGCTACCGTCAC	GGAACAGCGGAAAGTACTCG
Gstm4	M	AGGCTATGGATGTCTCCAATCAG	TCCAGGGAGCTGCTCCAA
Gpr37	M	ACTTGCCGTTATCTGGGTTG	ATACCACCAAAGCCTTGCAC
GPR37	H	ACGGTGACCAGTGATGACAA	CAGCAATGAGTTCCGACAGA
mGst2	M	CTGGCTGCAGTCTCTCTTCT	CCAGCCATCCACAGCATTAC
mGst3	M	ACGGGCATATGTTCAACTGC	GGGTCTCCTGTGTAGTAGCC
RSV L	V	GAACTCAGTGTAGGTAGAATGTTTGCA	TTCAGCTATCATTTTCTCTGCCAAT
RSV N	V	CATCTAGCAAATACACCATCCA	TTCTGCACATCATAATTAGGAGTATCA
Il-13	M	GTCCACACTCCATACCATGC	GATCTGTGTCTCTCCCTCTGA
Camp	M	CTTCAACCAGCAGTCCCTAGACA	TCCAGGTCCAGGAGACGGTA
IFNL1	H	CGCCTTGGAAGAGTCACTCA	GAAGCCTCAGGTCCCAATTC
IFNL2/3	H	AGTTCCGGGCCTGTATCCAG	GAGCCGGTACAGCCAATGGT
18s	H / M	GCAATTATTCCCCATGAACG	AGGGCCTCACTAAACCATCC

Table S1. Primer sequences used for quantitative PCR analysis of indicated genes. H, Human; M, mouse; V, virus.

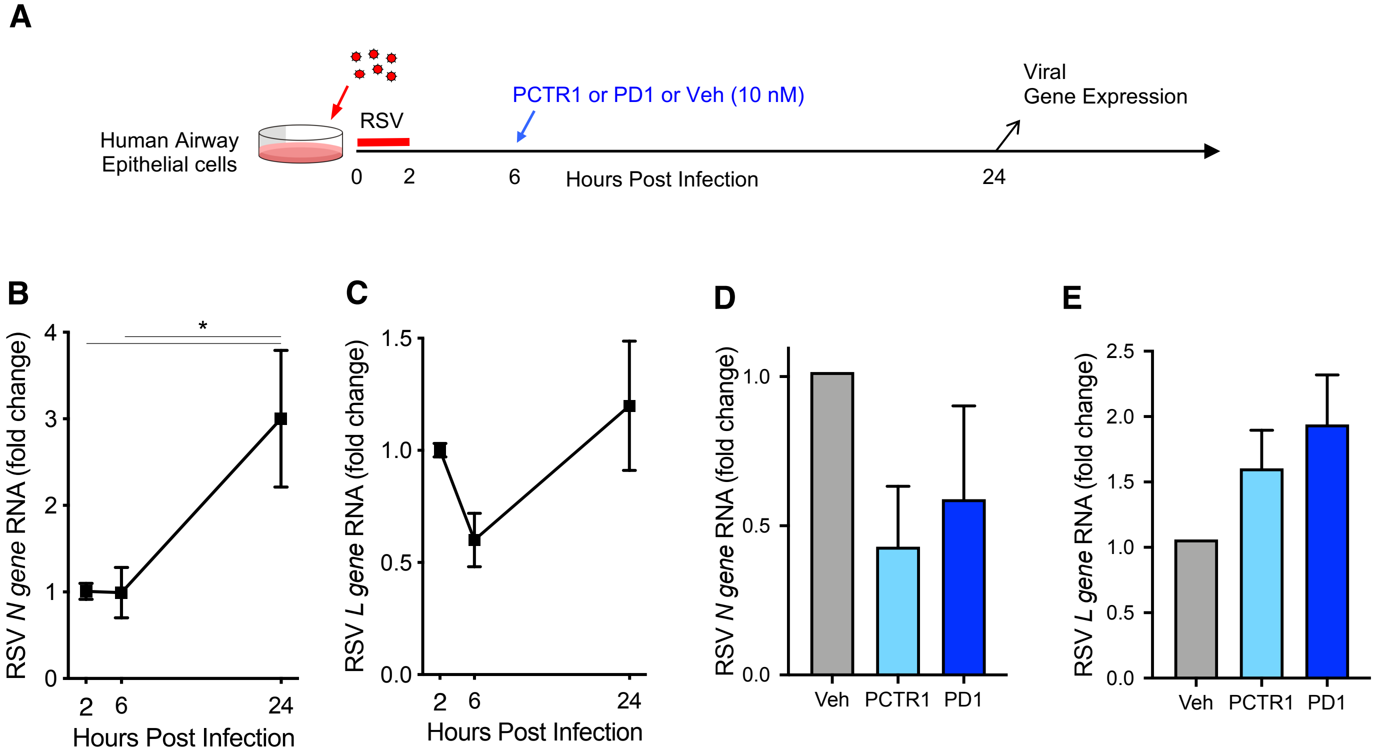

Figure S1. RSV infection propagates in human airway epithelial cells. (A) Schema of experimental RSV infection of Calu-3 human airway epithelial cells exposed to PCTR1 or PD1 (10 nM) or vehicle at 6 hours post-infection and measurement of viral gene expression. (B,C) Relative RNA expression of (B) RSV *N gene* and (C) RSV *L gene* viral transcripts over time in untreated cells. (D,E) Relative RNA expression of (D) RSV *N gene* and (E) RSV *L gene* after monolayer exposure to PCTR1, PD1 or vehicle. Values are mean of n = 2-3 per timepoint or n = 3-5 per exposure group ± SEM and include 2 separate experiments (D,E). * $p<0.05$ by one-way ANOVA with Holm-Sidak's multiple comparisons correction.

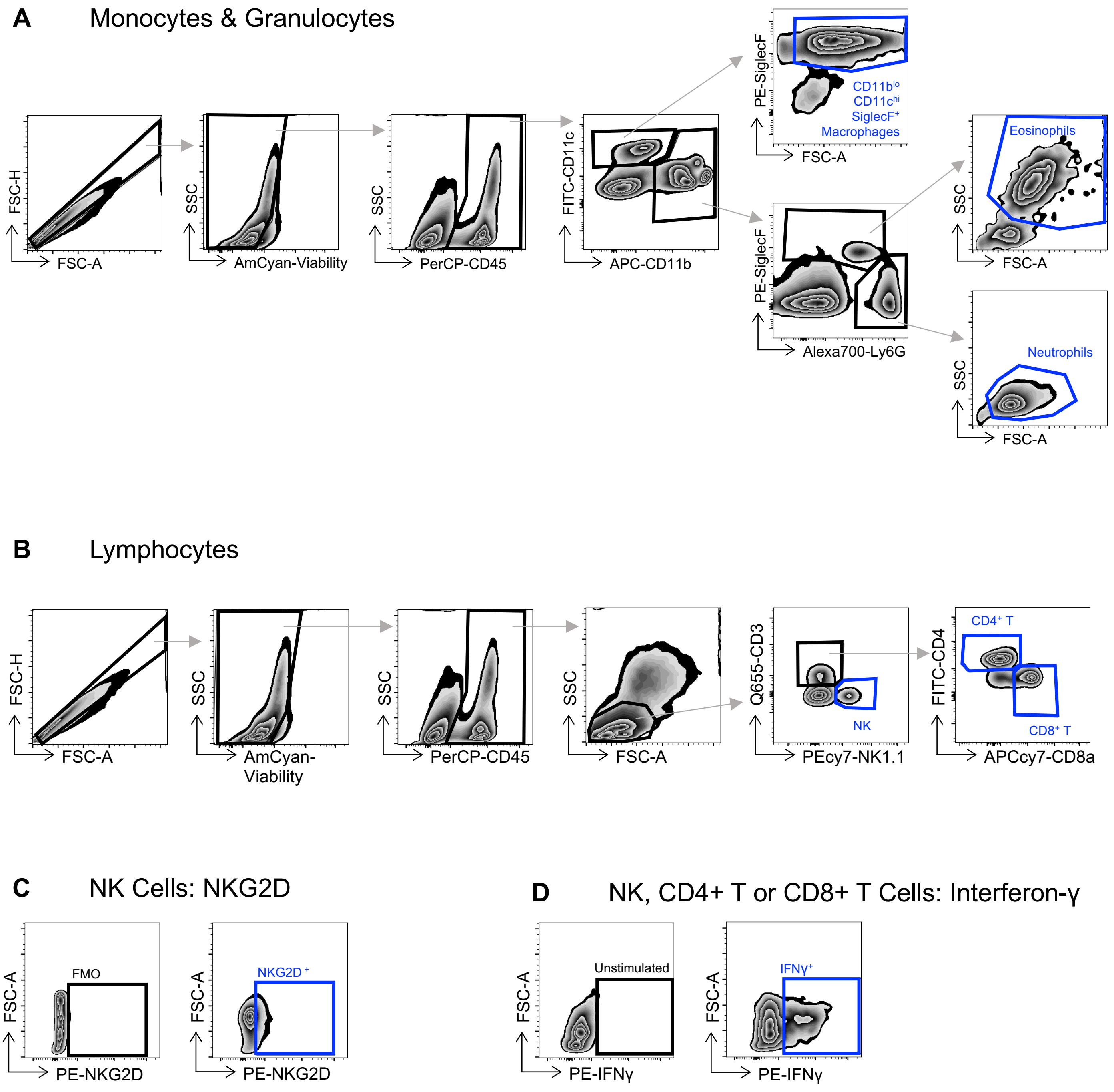

Figure S2. Flow cytometry gating strategies. Gating strategies for (A) Monocytes and granulocytes, (B) Lymphocytes. (C) NKG2D positivity with gate set by Fluorescence Minus One control, gated on NK cells. (D) Interferon-γ positivity with gate set by unstimulated control, gated separately on NK, $CD4^+$ T and $CD8^+$ T cells.

Mediator	Polarity	Retention Time (min)	Q1 (m/z)	Q3 (m/z)	Dwell Time (msec)	DP (V)	EP (V)	CE (V)	CXP (V)	Calibration Correlation Coefficient (r^2)	LLOD (pg)
**d_4-LTB_4**	Negative	14.23	339.2	197.1	36.697	-40	-10	-22	-12	0.998	0.07
**PD1**	Negative	13.99	359.2	153.1	31.676	-40	-10	-21	-12	0.997	0.04
**d_8-5S-HETE**	Negative	17.85	327.2	116.1	51.017	-40	-10	-17	-12	0.999	0.25
**17-HDHA**	Negative	17.24	343.2	245.1	37.492	-40	-10	-17	-12	0.999	0.25
**$^{13}C_2{}^{15}N$-PCTR1**	Positive	13.09	653.3	231.1	63.432	40	10	28	13	0.985	0.10
**$^{13}C_3{}^{15}N$-MCTR3**	Positive	13.77	468.2	191.1	74.762	40	10	23	13	0.988	0.50
**PCTR1**	Positive	13.10	650.3	231.1	63.353	40	10	28	13	0.995	0.10
**PCTR2**	Positive	12.46	521.3	231.1	113.926	40	10	23	13	0.998	0.10
**PCTR3**	Positive	13.75	464.3	231.1	83.650	40	10	23	13	0.996	0.50
**d_5-LTC_4**	Positive	13.89	631.3	194.1	88.314	40	9	28	15	0.995	0.04
**d_5-LTD_4**	Positive	13.08	502.3	194.1	65.908	40	9	28	13	0.999	0.25
**LTC_4**	Positive	13.93	626.3	189.1	93.295	40	10	28	13	0.996	0.04
**LTD_4**	Positive	13.09	497.3	189.1	65.908	40	10	23	13	0.996	0.50
**LTE_4**	Positive	14.38	440.3	189.1	200.00	40	10	23	13	0.999	0.25

Table S2. Sciex 6500⁺ Triple Quadrupole QTRAP Mass Spectrometer Settings. Columns indicate mediator name, polarity, retention time (min), Q1 (*m/z*), Q3 (*m/z*), dwell time (msec), declustering potential DP (V), entrance potential EP (V), collision energy CE (V), collision cell exit potential CXP (V), calibration correlation coefficient (r^2), and lower limit of detection (LLOD) in pg.

	Sciex ExionLC	
Column	Kinetex Polar C18<br>(100 mm X 4.6 mm X 2.6 μm)<br>Phenomenex	
Solvent A	Water (0.1% formic acid)	
Solvent B	Methanol (0.1% formic acid)	
Flow Rate	0.5 mL/min	
Gradient	Time (min)	Solvent B (%)
	0.1	45
	2.0	45
	16.5	80
	16.6	98
	18.5	98
	18.6	10
	20.5	End
	Sciex 6500⁺ Triple Quadrupole QTRAP<br>(Low Mass Mode)	
	MRM and EPI Mode	
Curtain Gas	30.0	
Collison Gas	12.0	
Ion Spray Voltage (V)	-4200 (+5200.0)	
Temperature (˚C)	520.0	
Ion Source Gas 1 (psi)	85.0	
Ion Source Gas 2 (psi)	50.0	

Table S3. Sciex ExionLC, MRM, and EPI Settings. Information is provided for the column, solvent system, flow rate (mL/min), gradient, and settings for multiple reaction monitoring (MRM) and enhanced product ion (EPI) on the Sciex 6500⁺ Triple Quadrupole QTRAP mass spectrometer set in low mass mode: curtain gas, collision gas, ion spray voltage (V), temperature (˚C), ion source gas 1 (psi, pounds per square inch), and ion source gas 2 (psi).

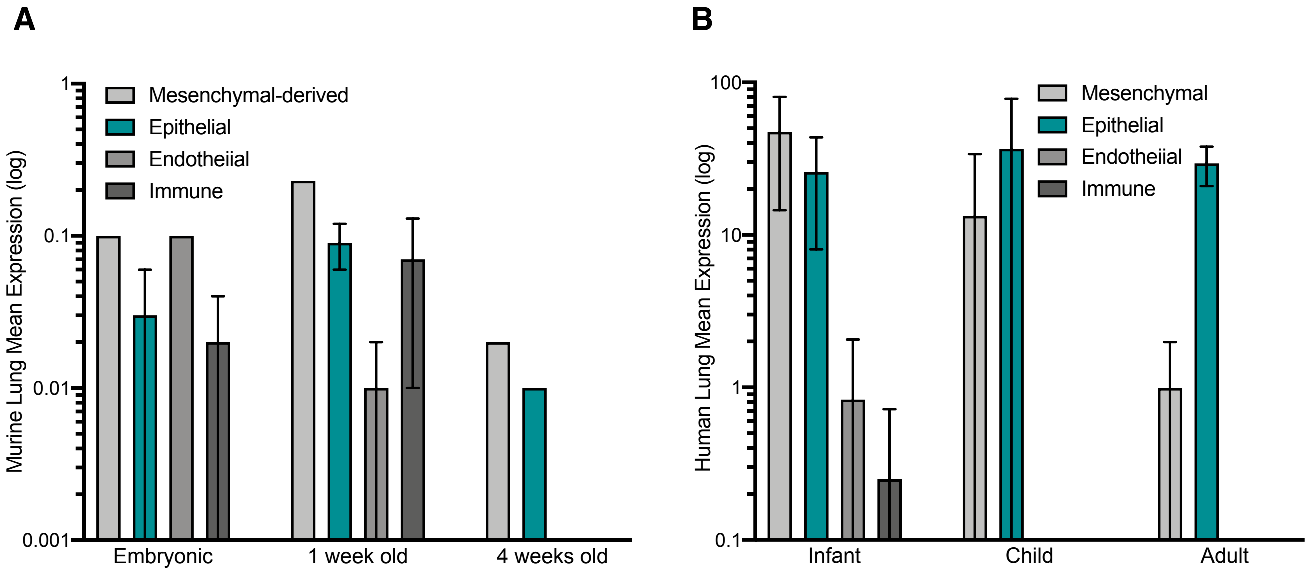

Figure S3. LungMAP data identifies protectin D1 receptor (GPR37*)* expression in mouse and human lung cells. *GPR37* RNA expression data from sorted lung cells in (A) mouse or (B) human lung tissue as reported in the public LungMAP database (https://lungmap.net). Bars indicate the mean gene expression and error as reported in the database. Embryonic refers to 16.5 days gestation; data for mice older than 28 days is not available.
